# A multi-epitope melanoma helper peptide vaccine durably increases Th1 cytokine production by responding lymphocytes

**DOI:** 10.1186/2051-1426-1-S1-P209

**Published:** 2013-11-07

**Authors:** Patrick M Dillon, Walter C Olson, Andrea Czarkowski, Gina R Petroni, Mark Smolkin, William W Grosh, Kimberly A Chianese-Bullock, Donna H Deacon, Craig L Slingluff

**Affiliations:** 1 Department of Medicine, University of Virginia, Charlottesville, VA, USA; 2 Department of Surgery, University of Virginia, Charlottesville, VA, USA; 3 Department of Public Health Sciences, University of Virginia, Charlottesville, VA, USA

## Background

Cancers produce soluble and cell-associated molecules that can suppress or alter antitumor immunity. Preclinical studies suggest the disease burden may alter the cytokine profile of helper T cell responses to cancer antigens. We studied cytokine production by helper T cells responding to vaccination with 6 melanoma helper peptides (6MHP) in blood and lymph nodes. We describe here the cytokine responses as change from baseline.

## Experimental design

Thirty-nine patients with stage IIIB-IV melanoma received a 6MHP vaccine. Antigen-reactive T cells from blood and draining lymph nodes were exposed to antigen, and supernatants (days 2 and 5) were assayed by cytokine bead arrays for Th1 and Th2 cytokines.

## Results

The greatest absolute cytokine responses detected were the levels of IFN-γ and IL-5, though high levels of IL-2 were also produced early by lymph node-derived lymphocytes. Lymphocytes from patients with clinically measurable disease produced similar levels of total cytokine as patients with no evidence of disease (NED). For both disease statuses, the cytokine profile was Th1-dominant in most patients. Total cytokine production and individual cytokine levels varied significantly among patients and varied over time but persisted up to nine months post-vaccine.

## Conclusions

The MHC class II-associated peptides used in this study induced helper T cells with a Th1-biased cytokine response in both PBMC and sentinel immunized nodes in most patients. Patients with or without measurable disease can mount a Th1 dominant response to these peptides, which may persist long after the vaccination sequence. Future studies are needed to test newer vaccine adjuvants in combination with these peptides.

